# Investigations of Cavitation Erosion and Corrosion Behavior of Flame-Sprayed NiCrBSi/WC-12Co Composite Coatings

**DOI:** 10.3390/ma15082943

**Published:** 2022-04-18

**Authors:** Costel-Relu Ciubotariu, Doina Frunzaverde, Gabriela Marginean

**Affiliations:** 1 Department of Engineering Science, Faculty of Engineering, Babeș-Bolyai University, Square Traian Vuia 1–4, 320085 Resita, Romania; relu.ciubotariu@ubbcluj.ro (C.-R.C.); doina.frunzaverde@ubbcluj.ro (D.F.); 2 Department of Materials Science and Testing, Westphalian University of Applied Sciences Gelsenkirchen Bocholt Recklinghausen, Neidenburger Str. 43, 45897 Gelsenkirchen, Germany

**Keywords:** corrosion, self-fluxing alloys, NiCrBSi, WC-12Co, cavitation, hard metals

## Abstract

Flame-sprayed NiCrBSi/WC-12Co composite coatings were deposited in different ratios on the surface of stainless steel. Oxyacetylene flame remelting treatment was applied to surfaces for refinement of the morphology of the layers and improvement of the coating/substrate adhesion. The performance of the coated specimens to cavitation erosion and electrochemical corrosion was evaluated by an ultrasonic vibratory method and, respectively, by polarization measurements. The microstructure was investigated by means of scanning electron microscopy (SEM) combined with energy dispersive X-ray analysis (EDX). The obtained results demonstrated that the addition of 15 wt.% WC-12Co to the self-fluxing alloy improves the resistance to cavitation erosion (the terminal erosion rate (Vs) decreased with 15% related to that of the NiCrBSi coating) without influencing the good corrosion resistance in NaCl solution. However, a further increase in WC-Co content led to a deterioration of these coating properties (the Vs has doubled related to that of the NiCrBSi coating). Moreover, the corrosion behavior of the latter composite coating was negatively influenced, a fact confirmed by increased values for the corrosion current density (i_corr_). Based on the achieved experimental results, one may summarize that NiCrBSi/WC-Co composite coatings are able to increase the life cycle of expensive, high-performance components exposed to severe cavitation conditions.

## 1. Introduction

The harsh environments encountered in different industrial domains expose mechanical components to extreme working conditions. Thus, parts of the equipment and structures used in fields, such as power generation, mining, shipbuilding, automotive, and aerospace, are subjected to severe surface degradation because of wear or/and corrosion, often followed by the failure of the entire components and the corresponding costs for downtime and replacement or repair of the degraded part [1,2,3,4].

Therefore, surface protection through wear- and/or corrosion-resistant coatings is considered to be an economical and effective method to prolong the service life of metallic components [1,2,4,5]. Among numerous layer-deposition methods, thermal spraying has been successfully used for long-term surface protection. Including various techniques, such as flame spraying, detonation spraying, wire arc spraying, high velocity oxy-fuel spraying (HVOF), and high velocity air fuel (HVAF) spraying, thermal spraying is a versatile technology, which can be used to deposit on various solid substrates high-quality coatings, with thicknesses ranging from a few microns to several millimeters, consisting of metals, ceramics, or combinations of these materials [2,4,5]. 

Some of these coatings require, depending on the feedstock material and deposition technique, particular post-treatment in order to enhance their quality and the adhesion to the metallic substrate and thereby their performance [2,6,7,8,9,10]. An impressive volume of research has been carried out during the last two decades to develop and optimize the material compositions used as feedstock for thermal-sprayed coatings aiming to obtain better wear and/or corrosion behavior of the deposited layers. 

Carbide-based materials with metallic binders (carbide-based cermets) are extensively used for numerous engineering applications to improve the wear resistance of the components, and it is assumed that their wear performance mostly depends on the carbide content, the carbide particle size, and their bond strength with the matrix [2]. While the ceramic component (WC, Cr_3_C_2_, TiC, ZrC, SiC, Mo_2_C) contributes to the hardness of the coating, the metallic binder (Co, Ni, Co-Cr, and Ni-Cr) ensures the necessary toughness of the coating [2,11,12]. Among the carbide-based cermets, the most popular in applications where good wear resistance is required are the WC-Co composites (WC-10Co, WC-12Co, and WC-17Co) [13] due to the high hardness, corrosion, and wear resistance of the tungsten carbide [14] and its excellent bonding strength to the tough Co matrix [2,11,12]. Particularly, regarding the wear behavior of the WC-12Co coatings, previous researchers reported that it shows good sliding wear resistance [11,15], abrasion wear resistance [15], erosive wear resistance [15], as well as cavitation erosion resistance [12,16,17,18]. One of the drawbacks of the Co binder for WC is the fact that, although it ensures coatings with outstanding wear resistance, the corrosion behavior does not meet the same expectations [2,13,17,19]. Therefore, to obtain WC-based coatings with better corrosion and oxidation resistance, the matrix phase may be completely or partially replaced with other materials such as Ni, Cr, or Ni-Cr [2]. 

On the other hand, excellent protection against corrosion can be offered by nickel-based, self-fluxing alloys, such as NiCrBSi, NiCrBSiMo, and NiFeCrBSi [4]. Usually, they are deposited by flame spraying, one of the oldest thermal-spraying techniques, which is simple, versatile, and cost-effective and can be easily employed not only for processing of new coatings but also for repairing of damaged and worn components [4,20,21]. As it produces porous coatings with relatively poor adhesion to the substrate and micro-cracks and lamellar boundaries resulting during the deposition of unmelted or semimelted particles, post-treatments can be applied by flame remelting or other methods aiming to increase the bonding with the base material and the homogeneity of the layer as well as to reduce its porosity and thereby enhance the coating properties [8,9,10,20,21,22,23,24,25,26,27]. Previous research has reported the good protection against corrosion combined with reasonable wear and friction performance offered by the flame-sprayed and flame-post-treated NiCrBSi coatings [7,8,21,25,27,28]. Moreover, NiCrBSi coatings are a less-expensive alternative to other materials, such as cermet powders, which are extremely cost-intensive [27].

Although NiCrBSi coatings are successfully used to improve corrosion resistance, they sometimes do not offer the desired wear resistance, especially in comparison with ceramic materials, as the adhesive wear of NiCrBSi coatings can lead to severe material removal despite having a relatively high hardness [14]. On the other hand, their low melting temperature makes them attractive for use as a primary binder to efficiently wet, adhere, and bind the reinforcing particles, so previous studies have been conducted to improve the wear resistance of this coating with the addition of hard carbides, such as WC [14,29,30], particularly obtained by mixtures of NiCrBSi powders with different amounts of WC-12Co powders. They report about the positive effect of the reinforcement of the NiCrBSi self-fluxing alloy by tungsten carbides in terms of improved hardness and increased wear resistance of the coating [14,27,28,31,32,33,34,35] as well as about the advantage over WC-Co or WC-CoCr coatings in applications where a more ductile coating is required [36]. 

Nevertheless, different conclusions have been drawn regarding the optimal ratio of WC and, respectively, WC-Co that ensures the best properties of the composite coating. While in [14], it was shown that the wear rate decreased with increasing content of WC-12Co from with 30, 40, and 50 wt.% for laser-cladded NiCrBSi composites, the addition of amounts exceeding 30% WC in the coating surface was found to have no significant influence on the wear resistance in case of coatings obtained also by laser cladding [34]. Supersonic plasma-sprayed NiCrBSi/WC-12Co coatings showed the best results for ratios at about 15 wt.% WC-12Co [32], whereas flame-sprayed coatings exhibited the best wear resistance for 25 wt.% WC-12Co addition to the NiCrBSi feedstock [35]. These different results may be caused by the different chemical compositions of the feedstock powders, the different coating methods applied, and/or the different equipment and deposition parameters applied.

However, only few researchers have focused on the issue regarding the effect of the amount of WC-12Co cermet added to the NiCrBSi self-fluxing alloy in order to enhance the wear resistance of the resulting composite coatings [32]. Furthermore, reports about the cavitation erosion resistance of NiCrBSi/WC-12Co composites are extremely rare. Only [37] partially touched the topic and carried out electrochemical corrosion and cavitation tests in 3.5 wt.% NaCl solution on WC-NiCrBSi coatings obtained by different vacuum-cladding technologies.

Surface damages caused by fatigue, corrosion, and wear, in particular cavitation erosion, are among the main causes that lead to the failure of hydropower plant components [7]. In spite of continuous design improvement, the use of high-quality stainless steels for component fabrication and the development of protective coatings for hydraulic turbine components exposed to severe cavitation, sometimes, the level of cavitation erosion is still unacceptable. Work-hardening austenitic stainless steels [38], ceramic-particle-reinforced polymers [39,40], or cermet coatings [2,12,16,17,18,19] are nowadays used in hydro-turbines to mitigate the degradation of the components by abrasion and cavitation. However, if the layers are not also corrosion-resistant, their protective properties can be lost after short operation times [19]. Therefore, for such applications, the use of flame-sprayed and flame-remelted cavitation and corrosion-resistive NiCrBSi/WC-12Co coatings could be an acceptable alternative for the protection of new components as well as for repair operations.

Based on the above mentioned, we aimed to point out the effect of the addition of WC-12Co to the NiCrBSi self-fluxing alloy on the microstructure, the hardness, the corrosion behavior, and the cavitation resistance of the resulting composite coatings. Therefore, for the reinforcement of the NiCrBSi coating, two ratios of cermet powder were used, respectively: 15 wt.% and 30 wt.% WC-12Co. The reference self-fluxing coating as well as the two composite coatings were deposited by flame spraying and were subsequently flame remelted. The surfaces of the deposited coatings were exposed to cavitation and to electrochemical corrosion in NaCl solution. The structural characteristics of the layers were investigated by scanning electron microscopy (SEM), and the qualitative chemical composition of the different structural components was evaluated by energy dispersive X-ray analysis (EDX). The influence of the reinforcing material on the microhardness of the composite coatings was determined and compared with that of the reference coating.

## 2. Materials and Methods

The reference coating was obtained by flame spraying of the NiCrBSi −106 + 45 µm feedstock powder (M-772.91 from FST Company, Duiven, The Netherlands/Cr, 10.0%; Fe, 2.5%; Si, 3.1%; B, 2.1%; C, 0.4%; Ni, balance). Further, two composite coatings were produced from powder mixtures of the self-fluxing alloy (SFA) with addition of 15 and, respectively, 30 wt.% WC-12Co powder −45 + 15 µm (no. 80.71.1 from GTV Company, Luckenbach, Germany). The coatings were deposited on martensitic stainless-steel substrates (X3CrNiMo13-4). Table 1 presents the sample labeling used in this research work depending on the ratio between the two types of powders. 

Prior to spraying, a sand-blasting machine was used to roughen the surface of the stainless-steel substrate. During the thermal-spraying process, the powder becomes liquid and deforms to impact, but the obtained coatings do not have a very dense microstructure. Therefore, flame remelting using an oxyacetylene gas process with a neutral stoichiometry was applied to refine the coating morphology, to reduce the porosity level, and to enhance the coating/substrate adhesion. The main parameters associated with the flame spraying process were as follows: oxygen flow 1.6 m^3^/h; acetylene flow 0.85 m^3^/h; spray distance 200 mm. After spraying, the coated material was fused with oxy-acetylene flame at 1000 ± 10 °C. 

**Figure 1:**
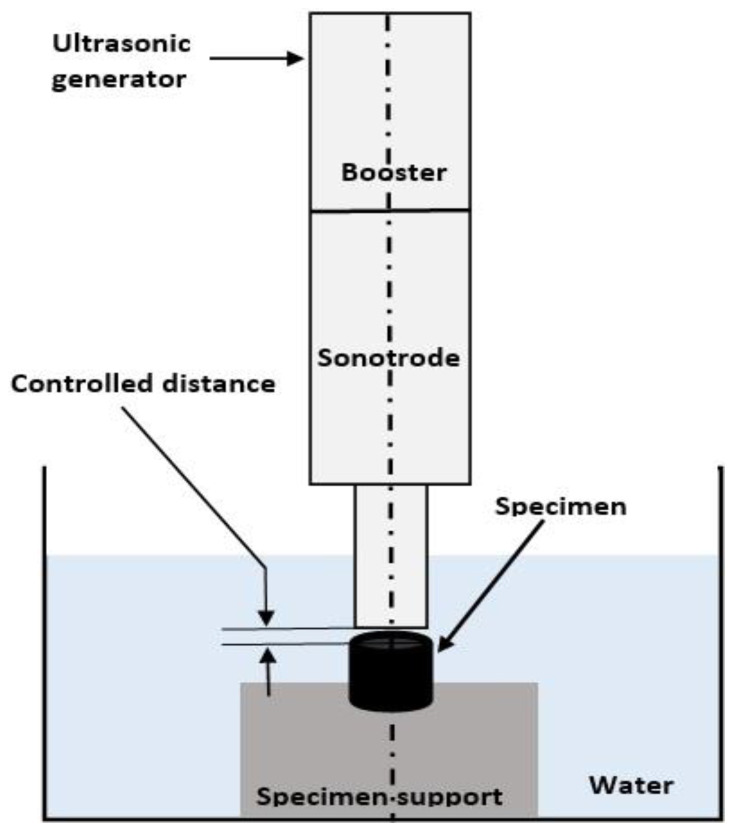
Schematic representation of the indirect cavitation method according to ASTM G-32 [41].

The hardness measurements were performed on cross-section specimens using a Zwick tester of type Z3.2A (ZwickRoell GmbH & Co. KG, Ulm, Germany). The Vickers hardness was determined by applying a 0.3 kgf load for 15 s. Electrochemical measurements were carried out to compare the corrosion resistance of the composite coatings with that of the reference NiCrBSi coating. Polarization curves (three measurements for each sample) were recorded in the positive potential direction at room temperature in a 3.5 wt.% NaCl solution with a three-electrodes cell using a saturated calomel electrode (SCE) as reference and platinum as auxiliary electrode. The applied potential was varied between −1000 mV and +1000 mV using a scanning rate of 10 mV/min. For the cavitation tests performed within this study, the vibratory indirect method was used with the specimen fixed and fully immersed in the liquid, as shown in Figure 1. 

The equipment used for determinations was a Telsonic DG-2000-2 Electronic Ultrasound Generator (Telsonic AG, Bronschhofen/SG, Switzerland). The resonance frequency of the oscillator was 20 ± 0.5 kHz, and the double (peak-to-peak) amplitude of the vibrating sonotrode was 50 μm. The test liquid was de-ionized water kept at room temperature with the aid of a control device and a water-cooling system. For each new test specimen, the fluid vessel was cleaned and refilled with fresh liquid. The distance between the vibrating sonotrode and the test specimen was controlled, as recommended in other research [38,42]. 

The specimens were tested for a total duration of 1800 min each, under the same conditions. The entire time interval was divided into 67 periods for performing the specimens weighing with a precision balance (five decimal places) after cleaning with acetone and subsequently air-drying. The test results are expressed by using the values of the progressive mass loss (∆m, also known as cavitation erosion) due to continued exposure to cavitation. The average value of ∆m was determined from the results obtained by measuring three samples for each type of coating. Further relevant values considered for the evaluation of the cavitation resistance of the three tested coatings were the cumulative erosion (CE) and, respectively, the terminal erosion rate in the stabilization stage (Vs) [41]. 

## 3. Results

### 3.1. Microstructural Characterization of the Coatings

**Figure 2:**
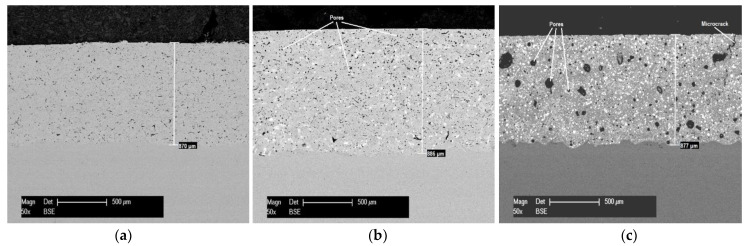
Cross-section SEM micrographs of the coatings: (**a**) SFA; (**b**) 85/15; (**c**) 70/30.

**Figure 3:**
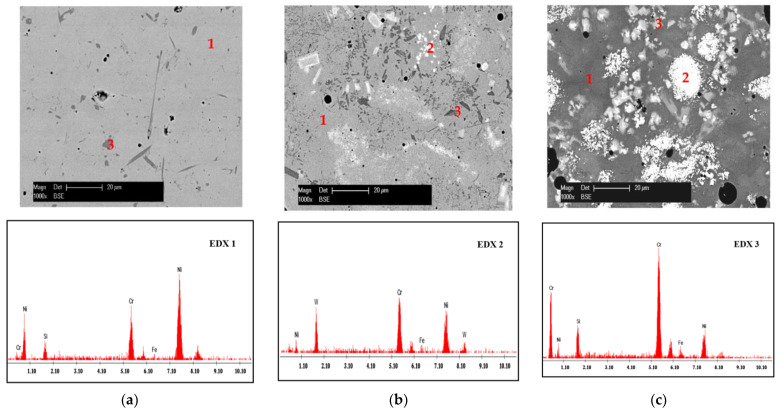
Cross-section SEM micrographs of the coatings at higher magnification combined with EDX analysis: (**a**) SFA; (**b**) 85/15; (**c**) 70/30.

The characteristics of the coatings concerning the morphology, microstructure, and distribution of phases with different chemical composition are presented in the cross-section SEM micrographs and, respectively, EDX spectra, shown in Figure 2 and Figure 3. The cross-section SEM micrograph of the 70/30 coating (Figure 2c) displays a higher degree of porosity and a slight tendency toward cracking in comparison with the other two coatings. 

Moreover, a further difference between the reference SFA coating and the composite coatings with 15 wt.% and, respectively, 30 wt.% hard particles of WC-12Co is the ratio of phases (metal matrix, carbides, or silicides). EDX analysis was performed for each gray-scale region corresponding to a particular phase from the microstructure. The analyzed regions were marked with the numbers 1, 2, and 3 and the corresponding EDX spectra with, respectively, EDX 1, EDX 2, and EDX 3 (Figure 3). As revealed by the results, the Ni-rich regions (1) are representing the metallic matrix, the W-rich particles (2) are constituents originated from the cermet powder, and the Cr/Si-rich phases (3) are silicides.

### 3.2. Microhardness Tests

**Figure 4:**
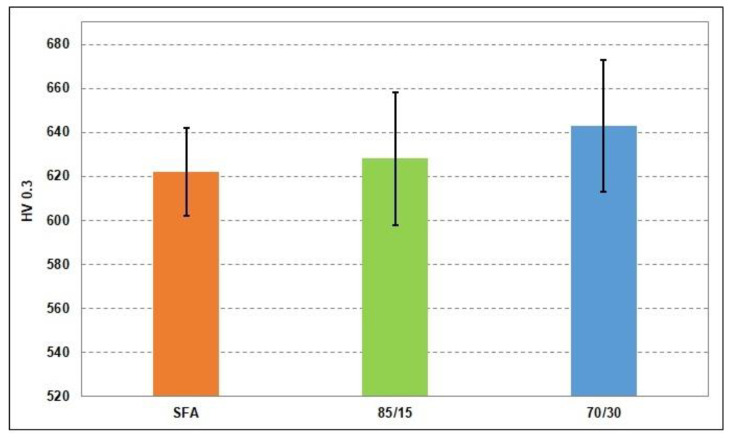
Microhardness values of the coatings.

Figure 4 shows the microhardness values calculated as the average of 10 indentations along the cross-section of the specimens. The standard deviation for the average value of the microhardness differs depending on the degree of heterogeneous phase distribution, respectively, on the local porosity. 

The sample with 30 wt.% WC-12Co exhibited slightly higher microhardness values in comparison with that of the reference SFA sample. It is attributed to the strengthening effect of reinforcements (>1000 HV), but the average values are not as high as expected. This fact correlates very well with the porosity of coating 70/30, which is significantly higher in comparison with that of the other two coatings. A general evaluation of the average hardness values of the composite coatings led to the conclusion that the process parameters must be further improved in order to achieve reliable results.

### 3.3. Corrosion Behavior

**Figure 5:**
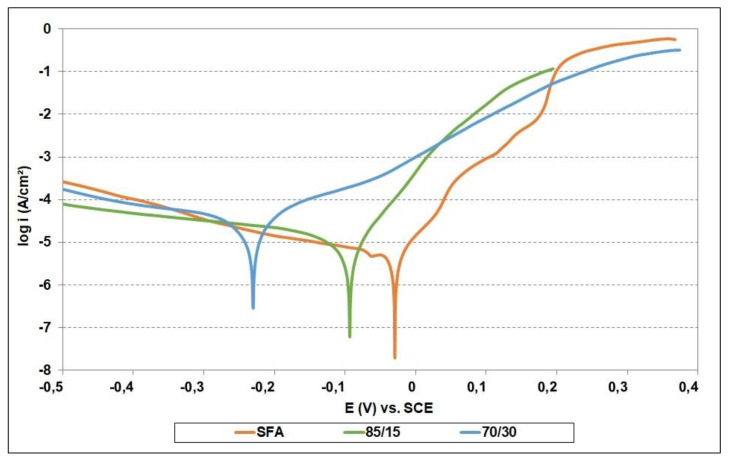
Potentiodynamic polarization curves of the coatings.

The values for the corrosion potential (E_corr_), the corrosion current density (i_corr_), and the corrosion rate (v_corr_) presented in Table 2 were determined following the Tafel extrapolation (Figure 5) of the polarization curves. Corrosion-potential values are directly associated with the chemical composition of the tested alloy, mainly with the elements responsible for corrosion resistance, such as Ni, Cr, or Co. The increase in the hard phase content of the self-fluxing matrix led to a slight shift of the E_corr_ values to the cathodic domain (to the left), indicating a less noble behavior. 

The values of the corrosion current densities show a slight increase for the 85/15 composite coating compared to the values determined for the reference SFA coating, a fact that correlates with the amount of internal porosity of the composite coating 85/15 (see Figure 2b). The same behavior was not observed for the 70/30 coating, which revealed considerable higher values of the current density almost six times higher in comparison with that of the SFA coating. These observations are based on the potential difference created along the phase boundary with the cermet particles (micro-galvanic regions) together with the presence of large pores and microcracks (see Figure 2c).

### 3.4. Cavitation Erosion Behavior

**Figure 6:**
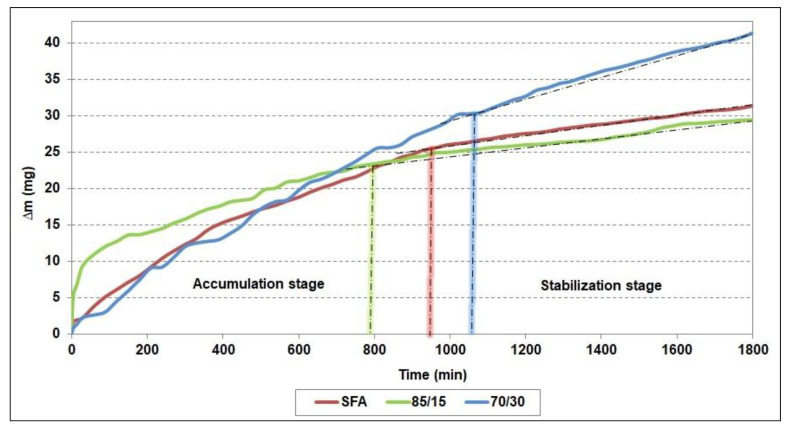
Progressive mass loss during exposure to cavitation.

The evolution of the progressive mass loss during the test for the SFA and the two composite coatings (85/15, 70/30) are graphically presented in Figure 6. According to [32], the erosion behavior of the investigated coatings was evaluated considering the erosion rate in the stabilization stage. This parameter can be considered independent toward to roughness of the eroded surface and to initial material loss in the accumulation stage. 

Starting from this idea, the terminal erosion rate (Vs) was calculated for all tested surfaces by the ratio between the mass variation in the stabilization stage and the corresponding time interval. The values of the cumulative erosion (CE) after 1800 min of exposure to cavitation and, respectively, the terminal erosion rate in the stabilization stage (Vs) for the SFA coating and the two composite coatings (85/15 and 70/30) are displayed in Table 3. 

Analyzing the information from Figure 6 and Table 3, one may observe that an addition of 15 wt.% WC-12Co to the NiCrBSi matrix led to an improvement of its cavitation behavior since CE and Vs showed lower values than in case of the SFA. It should also be noted that the 85/15 composite coating reached the stabilization stage after 750 min of exposure to cavitation, 150 min earlier than the SFA. Moreover, the terminal erosion rate of the 85/15 coating was about 15% lower than that of the SFA.

On the other hand, an increased content of WC-12Co led, in the case of the 70/30 coating, to a noticeable worsening of the cavitation behavior compared both to the SFA and the 85/15 composite samples. In this case, the stabilization stage was reached after 1020 h of cavitation, and CE and Vs were considerably higher. This may be explained by the higher content in hard W-rich phases (Figure 3c, constituent type 2) of the 70/30 coating, which suffered a preferential pull-out phenomenon during the cavitation erosion along the interface to the matrix.

## 4. Conclusions

NiCrBSi/WC-12Co composite coatings with different ratios were obtained by flame spraying and subsequent oxyacetylene flame remelting. The NiBCrSi coating had a denser structure in comparison with that of the composite coatings. The degree of porosity increased with the amount of WC-12Co (e.g., 70/30). The ceramic hard particles were uniformly distributed in the NiCrBSi alloy matrix, exhibiting a good adhesion along their interface.

The average microhardness of the 70/30 composite coating was higher than that of the 85/15 composite coating and, respectively, the SFA alloy but did not reach the expected hardness in relation to the hard metal amount. There are at least two reasons that negatively influenced the determined microhardness, namely the high degree of porosity and the tendency to crack formation.

The 85/15 composite coating exhibited the best cavitation behavior, without a significant deterioration of the corrosion resistance in the chlorine-containing aqueous solution. The good bonding between the WC-12Co hard phase and the NiCrBSi alloy matrix and the moderate degree of porosity had an important contribution to the properties of this composite coating. A further increase of the WC-12Co amount in the NiCrBSi alloy led to a significant decrease of both the corrosion and the cavitation resistance of the respective composite materials.

Flame-sprayed NiCrBSi/WC-12Co composite coatings, subsequently remelted by oxyacetylene flame, are suitable for the protection of components against corrosion and cavitation. However, the amount of hard particles has to be further optimized in close correlation with the process parameters.

## Figures and Tables

**Table 1:** Sample designation in respect to the powder mixture.

Sample Designation	Powder Mixture (in wt.%)
85/15	85% NiCrBSi + 15% WC-12Co
70/30	70% NiCrBSi + 30% WC-12Co

**Table 2:** Corrosion test results.

Sample	E_corr_ (mV)	i_corr_ (µA/cm^2^)	v_corr_ (µm/year)
SFA	−27.0 ± 2.0	4.49 ± 0.5	52.53 ± 3.0
85/15	−91.7 ± 5.0	5.95 ± 0.5	69.62 ± 3.2
70/30	−227.6 ± 5.0	28.01 ± 1.0	187.60 ± 6.5

**Table 3:** Values of the cumulative erosion (CE) after 1800 min of exposure to cavitation and of the terminal erosion rate (Vs).

Parameter	SFA	85/15	70/30
CE (mg)	31.40 ± 0.4	29.41 ± 0.6	41.39 ± 1.2
Vs × 10^−3^ (mg/min)	7.44 ± 0.2	6.35 ± 0.2	14.38 ± 0.5

## Data Availability

The data reported in this study are available from the authors upon request.
